# Mapping the polymorphic transformation gateway vibration in crystalline 1,2,4,5-tetrabromobenzene†
†Electronic supplementary information (ESI) available. See DOI: 10.1039/c8sc03897j


**DOI:** 10.1039/c8sc03897j

**Published:** 2018-11-23

**Authors:** Adam J. Zaczek, Luca Catalano, Panče Naumov, Timothy M. Korter

**Affiliations:** a Department of Chemistry , Syracuse University , 1-014 Center for Science and Technology , Syracuse , New York 13244-4100 , USA . Email: tmkorter@syr.edu; b New York University Abu Dhabi , P.O. Box 129188 , Abu Dhabi , United Arab Emirates

## Abstract

The thermosalient behavior of 1,2,4,5-tetrabromobenzene (TBB) is related to a temperature-induced polymorphic structural change. The mechanism behind the phase transition has been investigated in this work using low-frequency (10–250 cm^–1^) Raman spectroscopy and solid-state density functional theory simulations. Careful adjustments of the probing laser power permitted thermal control of the polymorph populations and enabled high-quality Raman vibrational spectra to be obtained for both the β (low temperature) and γ (high temperature) forms of TBB. Numerous well-defined vibrational features appear in the Raman spectra of both polymorphs which could be assigned to specific motions of the solid-state TBB molecules. It was discovered that the lowest-frequency vibration at 15.5 cm^–1^ in β-TBB at 291 K is a rotational mode that functions as a gateway for inducing the polymorphic phase transition to γ-TBB, and serves as the initiating step in the storage of mechanical strain for subsequent macroscopic release. Computationally mapping the potential energy surface along this vibrational coordinate reveals that the two TBB polymorphs are separated by a 2.40 kJ mol^–1^ barrier and that γ-TBB exhibits an enhanced cohesion energy that stabilizes its structure.

## Abstract 2

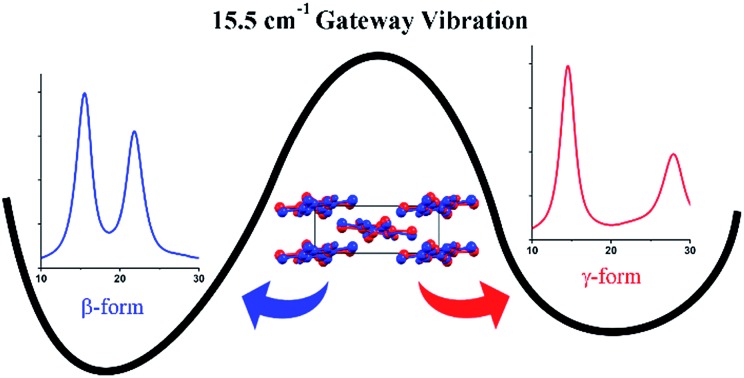
A single lattice vibration at 15.5 cm^–1^ serves as a gateway for the polymorphic conversion of thermosalient 1,2,4,5-tetrabromobenzene crystals.

## I. Introduction

The thermosalient effect is a phenomenon where a crystal undergoes a structural transformation due to changes in temperature, and this produces a physical “jumping” of the crystal.^1^–^3^ From an atom-level perspective, the physical motion corresponds with sudden yet small changes of the molecular structures and packing arrangements of the components comprising the crystalline solid. This unusual behavior has been attributed to material changes such as molecular dimerization (photosalient effect)^4^ and polymorph transformations^5^,^6^ (thermosalient effect). Given that this is often a reversible process^7^,^8^ controlled by varying sample temperature, the effect has attracted interest in the development of new energy transducing and actuating materials, such as those in electronics applications.^9^

One of the more well-studied thermosalient materials is 1,2,4,5-tetrabromobenzene (TBB).^10^–^13^ TBB is often used as a starting material in the organic syntheses of liquid crystals^14^ and photoconductive polymers.^15^ Below 307 K 12,16 (previously reported at 319 K in ref. 17 and 18), crystalline TBB naturally exists in its β polymorph, with the γ polymorph forming at higher temperatures. Single crystals of TBB are often twinned,^19^,^20^ and the crystals jump or split upon polymorph transformation, propelling themselves several centimeters. The jumping distances are largely dependent on the crystal size and resting face of the crystal, with large crystals having more pronounced jumping.^10^

**Fig. 1:**
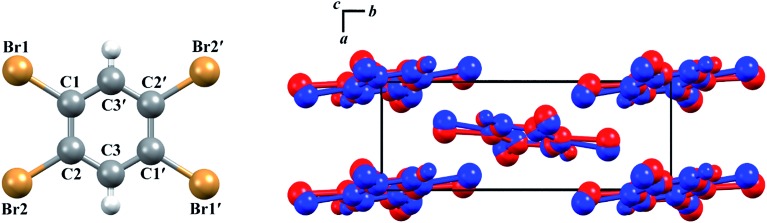
Molecular structure of a TBB molecule (left) and the packing differences between the two TBB polymorphs, β-TBB (blue) and γ-TBB (red).

The structures of both TBB polymorphs have been previously determined through single-crystal X-ray diffraction,^21^,^22^ revealing that both exist in the same space group (*P*2_1_/*n*) with similar unit cell dimensions, differing primarily through an intermolecular tilting of the TBB rings with respect to one another in the unit cell, as shown in Fig. 1. Because the polymorph structures are quite similar to one another and do not intuitively correspond to the significant structural changes that might be associated with thermosalient behavior, it warrants further analysis of the crystal structures, the intermolecular lattice vibrations, and the underlying mechanism responsible for the conversion between the solid forms.

Recent work has investigated the structural strain associated with the polymorph change that contributes to the thermosalient nature of TBB, and suggested that low-frequency vibrational modes near 43 cm^–1^ and 47 cm^–1^ are integral to the conversion.^12^ The importance of low-frequency vibrations for determining the properties of TBB crystals is consistent with the established relationship that exists between low-frequency lattice phonons and displacive/martensitic phase transformations as observed in other crystalline solids.^23^–^25^ In the current study, experimental low-frequency Raman spectral data (10–250 cm^–1^) was collected at various temperatures and combined with solid-state density functional theory (ss-DFT) simulations to provide clear assignments of the vibrational motions in this region. This granted the ability to uniquely identify a single mode as being the gateway vibration for β-TBB to γ-TBB conversion. An in-depth computational study of the vibrational energies, motions, and potential energy surfaces of six rotational modes present in the β-TBB crystals revealed that only the vibration observed at 15.5 cm^–1^ (at 291 K) is able to initiate the β → γ transformation. The combination of experimental Raman data and *ab initio* computational analyses enables the mechanism of the polymorph transformation to be understood and quantified in terms of relative energies and barriers to conversion.

## II. Methods

### a. Experimental details

TBB was purchased from Sigma-Aldrich and used without further purification. Sample composition was confirmed *via* powder X-ray diffraction, showing β-TBB. Raman spectra were obtained with an Ondax (Monrovia, CA, USA) THz-Raman spectrometer with a laser centered at 784.7 nm and coupled to an Andor (Concord, MA, USA) Shamrock 750 spectrograph with an Andor iDus 416 CCD. The data spanned ±250 cm^–1^ from the Rayleigh peak, with an effective spectral resolution of 0.6 cm^–1^. For room-temperature studies, the TBB sample was kept as a loose powder in a 10 mm diameter glass vial. The spectra shown here were averaged over 225 acquisitions, each with a 3 second exposure time. A range of laser power levels was used (2.5, 5.2, 13.1, 17.7, 74, and 115 mW) and controlled by a variable neutral density filter.

For cooled studies, the powder samples were compressed into free-standing pellets (13 mm diameter, ∼1 mm thick) using pure TBB and mounted in a liquid-nitrogen-cooled cryostat. With an applied pelleting pressure of 0.07 GPa, the sample disc showed β-TBB to be the major constituent, but with 0.14 GPa applied, γ-TBB was the major component. Additional pellets were made at increased pressures (0.20 and 0.28 GPa) to evaluate the influence on TBB stabilization and were examined only at 290 K. The laser power was kept to a minimum (13.1 mW) for all cooled pellet experiments to avoid unwanted polymorph conversion while maximizing the scattering signal.

### b. Computational details

All solid-state density functional theory (ss-DFT) calculations were performed using the CRYSTAL17 (ref. 26) software package. The Perdew–Burke–Ernzerhof (PBE) exchange and correlation functional^27^ was used with the def2-TZVP^28^ basis set. Grimme's D3 dispersion correction^29^,^30^ with an included Becke–Johnson dampening parameter^31^ was employed to treat weak long-range London dispersion forces between molecules.

Geometry optimizations were begun using previously published crystal structures for β-TBB^21^ and γ-TBB^22^ as initial starting points. To account for the effects of non-zero experimental temperatures on the unit cell dimensions in the calculations, atomic positions were allowed to optimize to their energetic minima within fixed lattice dimensions and space group symmetries determined by the reported single-crystal X-ray diffraction measurements. For β-TBB (the low-temperature form), a free optimization that did not constrain lattice dimensions was also run for comparison. An energetic convergence of Δ*E* < 10^–8^ hartree was used for all optimizations with root-mean-square thresholds (in atomic units) set to 1.0 × 10^–5^ for the gradients and 4.0 × 10^–5^ for the estimated displacements. Separate single-point energy calculations were performed on the optimized structures in order to determine the energy stability rankings of the polymorphs, and these were corrected for basis set superposition errors (BSSE) using the counterpoise method.^32^

Harmonic normal mode frequency analyses were executed on the structurally optimized TBB solids with a stricter energy convergence of Δ*E* < 10^–10^ hartree. Raman intensities were calculated by computing the Raman tensor utilizing a coupled-perturbed Hartree–Fock/Kohn–Sham approach.^33^,^34^ To accelerate convergence, a mixing of Fock/KS matrix derivatives^35^ was used. Experimental temperatures and laser excitation wavelength were also accounted for in the simulated intensities. Normal mode analyses formed the basis for constrained unit cell optimizations, using eigenvector-displaced molecular positions to determine the influence of specific lattice vibrations on the polymorphic stabilities.

For all calculations, truncation tolerances for the Coulomb and Hartree–Fock exchange integrals (keyword TOLINTEG) were set to 10^–12^, 10^–12^, 10^–12^, 10^–24^, 10^–48^ hartree. A sampling of 205 *k*-points was used in the irreducible Brillouin zone (keyword SHRINK = 9), and a predefined grid size of 366477 points was utilized (keyword XXLGRID).

## III. Results and discussion

### a. Raman spectroscopy of powder samples

**Fig. 2:**
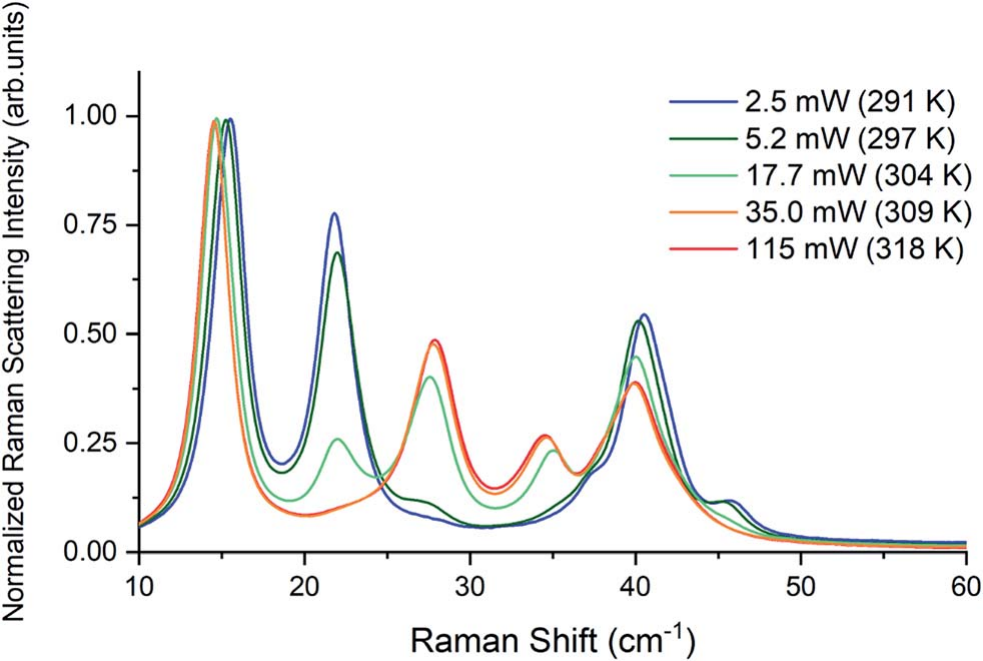
Low-frequency Raman spectra of TBB at varying applied laser powers.

Raman spectra of TBB powder were taken using a 784.7 nm laser with varying power levels, with the spectrum at the lowest power (2.5 mW) showing β-TBB, and the maximum power (115 mW) revealing γ-TBB to be present (Fig. 2). The spectral features of both forms were consistent with prior reports,^36^,^37^ aiding in their identification. The samples at the laser power extremes were found to be at least 96% polymorphically pure within the limits of detection. Polymorph changes occurred due to laser heating of the sample, with greater powers imparting more heat and causing γ-TBB formation. At intermediate powers, a mix of both polymorphs was observed, which may be attributed to uneven sample heating leading to localized partial polymorph conversion in the beam path. After approximately 35.0 mW, γ-TBB was observed as the clear majority product. No thermosalient crystal movement was observed in the powder sample during heating (likely due to the very small particle size), indicating that the spectra were representative of the same probed area each time.

**Fig. 3:**
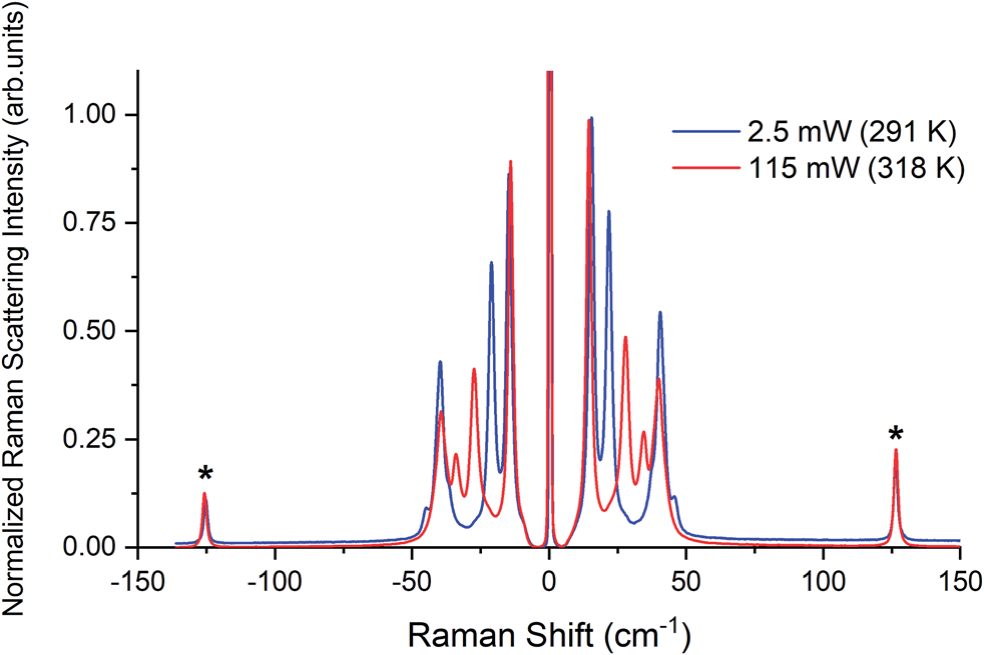
Raman spectra of β-TBB (blue) and γ-TBB (red) obtained from powder samples with peaks used for temperature analysis marked by asterisks (*).

It is possible to calculate the average temperature (*T*) of a sample at the laser focus directly from the Raman spectral data by utilizing the relative intensities of the anti-Stokes/Stokes scattering features and the following equation:1
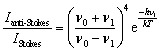
where *I* values originate from the intensities of an anti-Stokes and Stokes peak pair, *ν*_0_ is the frequency of the exciting light, *ν*_1_ is the frequency of the Raman shift, *h* is Planck's constant, and *k* is the Boltzmann constant. Temperatures were calculated using the spectral features at ±126.4 cm^–1^ from the Rayleigh peak, as these are single narrow features that are present in both TBB polymorphs, as shown in Fig. 3. The peaks were fit with a Lorentzian line shape, and the areas under the best fit curves were used in the anti-Stokes/Stokes ratio. All sample temperatures reported in this work have been determined in this way. The lowest applied power of 2.5 mW was found to produce a sample temperature of 291 ± 3 K, which is in agreement with the laboratory temperature of 290 K, and showed the spectrum of β-TBB. The highest laser power data (115 mW) showed that the sample had been heated to 318 ± 7 K and revealed the Raman spectrum of γ-TBB, in agreement with previous reports noting its dominance above 307 K.^12^

### b. Raman spectroscopy of pelleted samples

#### i. β-TBB

**Fig. 4:**
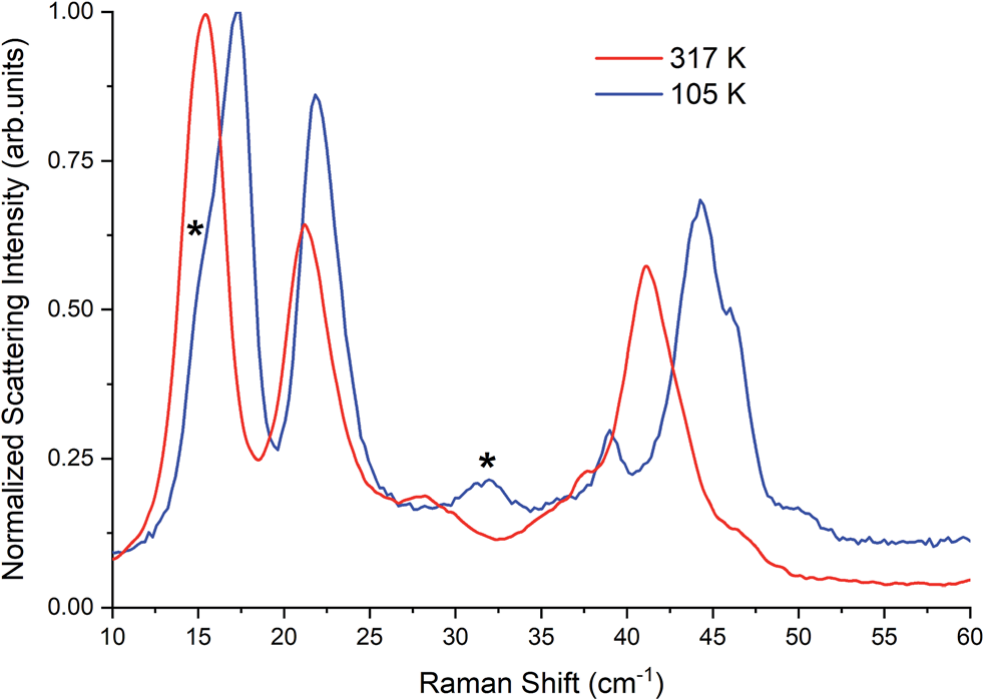
Raman spectrum of β-TBB at 317 K and 105 K. Contributions from γ-TBB in the 105 K spectrum are marked with asterisks (*).

A β-TBB pellet made from the application of 0.07 GPa was placed in a cryostat and studied both with liquid-nitrogen cooling and with no cooling. A low laser power (13.1 mW) was used in all cases to record the Raman spectra shown in Fig. 4 and at-sample temperatures of 317 ± 6 K (uncooled) and 105 ± 4 K (cooled) were determined. The pellet spectra match with the corresponding Raman spectra of the powder samples, with the only differences (referring to the 105 K data) being a shoulder feature at 14.8 cm^–1^ and a peak at 32.2 cm^–1^ originating from a small amount of coexisting γ-TBB. The γ-TBB contributions have been identified through a direct comparison of the pellet data over a range of temperatures and compared to TBB powder samples (data provided in the ESI†). The unexpected presence of γ-TBB at 105 K may be due to transient heating upon pressure application during sample preparation, but it may also be from pressure-induced formation given that γ-TBB has been reported to be stable in nanopore structures.^10^ Similarly, the observation of the clear Raman features from β-TBB at 317 K is inconsistent with the reported transition temperature of 307 K, but it is apparently stabilized by the applied pelleting pressure. Using a linear combination of the powder spectra, it was found that the sample pellet created at 0.07 GPa contained approximately 90% β-TBB and 10% γ-TBB. While the presence of γ-TBB was initially undesirable, this result presented an opportunity since this demonstrated it could be stabilized and studied simultaneously with β-TBB at low temperatures.

#### ii. γ-TBB

**Fig. 5:**
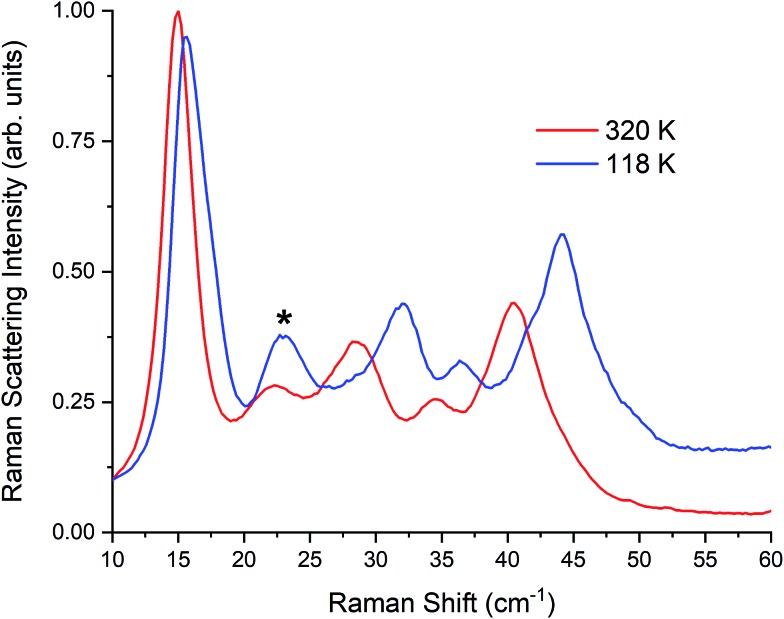
Raman spectra of γ-TBB at 320 K and 118 K. Contribution from β-TBB is marked in the 118 K spectrum with an asterisk (*).

To further test the stabilization of γ-TBB with pressure, another pellet was made by compressing to 0.14 GPa in an attempt to create a pellet with a higher γ-TBB concentration. The resulting sample was found to contain 80% γ-TBB, with the remainder being β-TBB. Further evidence that these peaks correspond to β-TBB is demonstrated in spectra at intermediate temperatures, provided in the ESI.† Remarkably, when this pellet was cooled to cryogenic temperatures (well below the temperature that γ-TBB should be stable), the spectral features indicated that no conversion to β-TBB had occurred. The temperature-dependent spectra for γ-TBB at 320 ± 7 K and 118 ± 4 K are shown in Fig. 5. This is the first time that γ-TBB has been stabilized at this low a temperature, although recent work has shown that γ-TBB nanocrystals have been stabilized in anodic aluminum oxide nanopores to 183 K.^10^

Pressure clearly plays a role in TBB polymorph stabilization. To investigate this, additional pellets were made at varying pressures (0.03, 0.10, 0.20, and 0.28 GPa). While very little change was observed in the room-temperature Raman spectra at intermediate pressures, the 0.28 GPa pellet was found to contain an additional 5% γ-TBB (85% in total). Although this did not eliminate the β-TBB form completely, it showed that pressure increases do promote γ-TBB stabilization. It is worth noting that higher pressures (0.7 GPa) can cause formation of α-TBB,^38^ but the pressures used in the present study were not great enough to observe any trace of this polymorph.

### c. Solid-state density functional theory simulations

#### i. Structural optimizations

ss-DFT simulations were initially performed on the β-TBB polymorph. A full optimization of the unit cell dimensions and atomic positions produced excellent results (Table 1), with errors ≤0.15% in all cell parameters. However, when full optimizations were performed on γ-TBB, the structure immediately relaxed to the β-TBB form. The ss-DFT simulations are performed at 0 K where β-TBB is experimentally known to be more stable, so the conversion is not surprising. The two polymorphs exhibit the same space group symmetry (*P*2_1_/*n*) and differ only by a small intermolecular tilting of the rings. However, it is important to note that the γ-TBB crystal maintains its form and optimizes without issue (including having all positive frequency eigenvalues) if the unit cell volume is constrained and held constant at the 332 K experimental value. This indicates that both polymorph structures represent minima on the solid-state potential energy surface (at least in terms of enthalpy), but that thermal expansion and contraction of the unit cell plays an important role in defining polymorph stabilities.

**Table 1:** Comparison of the measured β-TBB crystalline dimensions and results from a fully optimized PBE-D3/def2-TZVP structure

	*a* (Å)	*b* (Å)	*c* (Å)	*β* (°)	Volume (Å^3^)
Experimental^21^ (100 K)	3.9235	10.4885	10.3675	100.367	419.675
Calculated	3.9241	10.5012	10.3559	100.516	419.580
Error (%)	0.02	0.12	–0.11	0.15	–0.02

Given these results, optimization of γ-TBB was done with crystal lattice parameters held at experimentally observed values (332 K (ref. 22)) and the atoms were allowed to relax to an energetic minimum within these constraints. While no problems were encountered in the full optimization of the β-TBB structure, for a fair comparison to the fixed-lattice γ-TBB calculations, a fixed-lattice (100 K (ref. 21)) optimization was also performed on β-TBB. All energy and vibrational analyses presented in this work are based on the fixed-lattice calculations. Regardless of the constraints on the geometry optimizations, the molecular structures produced in the simulations agreed very well with experimental data, as shown by the calculated intramolecular distances and angles, as well as the intermolecular distances, shown in Table 2.

**Table 2:** Comparison of the experimental and calculated bond lengths (Å), intramolecular angles (°), dihedral angles (°), and intermolecular distances (Å) involving non-hydrogen atoms for β-TBB and γ-TBB

Intramolecular bond (Å)	β-TBB		γ-TBB	
	Experimental	Calculated	Experimental	Calculated
C1–C2	1.397	1.401	1.358	1.371
C1–C3′	1.401	1.396	1.420	1.406
C2–C3	1.387	1.395	1.372	1.399
C1–Br1	1.884	1.895	1.844	1.873
C2–Br2	1.883	1.896	1.917	1.904

**Angle (°)**				
C1–C2–C3	121.82	121.74	121.74	121.82
Br2–C2–C3	118.15	118.34	118.34	118.15
C1–C2–Br2	120.03	121.74	121.73	120.03
Br1–C1–C2	119.84	119.63	119.63	119.84

**Dihedral angle (°)**				
C3–C1–C2–C3	0.07	0.10	1.67	0.03
Br1–C2–C1–C3	179.58	179.53	177.74	179.48
Br1–C1–C2–Br2	0.36	0.55	1.07	0.87
Br2–C1–C2–C3	180.00	179.98	178.15	179.63

**Intermolecular bond (Å)**				
Br1···C3	3.740	3.734	4.158	4.182
Br2···C3	4.477	4.469	4.129	4.154

#### ii. Vibrational analysis

**Fig. 6:**
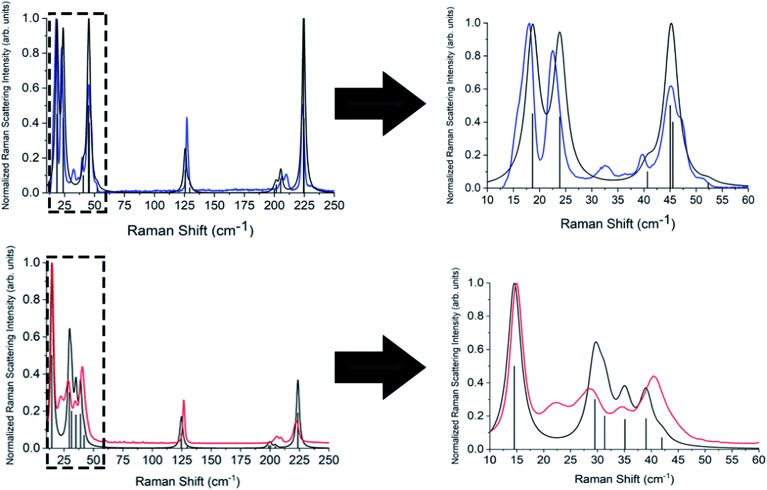
Experimental Raman spectra for β-TBB (blue) at 107 K and γ (red) at 318 K with their corresponding simulated (black) Raman spectra. The boxed areas in the lower frequency range (left) are shown expanded on the right.

Simulations of the Raman spectra of both TBB polymorphs were in excellent agreement with experimental observations (Fig. 6) and no scaling of the vibrational frequencies was applied. A complete list of all calculated vibrational modes is available in the ESI.† The β-TBB and γ-TBB simulations were compared to their corresponding 105 K and 320 K spectra, respectively, as these were closest to the temperatures of the reported crystallographic data. All Raman-active modes below 100 cm^–1^ represent rigid rotations of the TBB molecules in their lattice positions. This was determined through analysis of the normal mode eigenvector displaced structures, which yielded no significant changes in the intramolecular geometries of the TBB molecules (see ESI†). The peak at 126.4 cm^–1^ which had negligible shifting experimentally corresponds to an in-plane intramolecular bromine wagging (calculated at 125.6 cm^–1^ and 124.7 cm^–1^ in β-TBB and γ-TBB, respectively), while the peaks in the 190–250 cm^–1^ region originate from intramolecular ring torsions.

Complete lists of the experimental and calculated Raman-active modes for both TBB polymorphs below 250 cm^–1^ are shown in Tables 3 and 4. The β-TBB vibrational simulations are in excellent agreement with the current experimental results, with the only peaks not modeled arising from the aforementioned γ-TBB content. A recent study has presented a similar vibrational analysis of β-TBB,^12^ but it did not experimentally access the significant sub-40 cm^–1^ range. The peaks calculated for γ-TBB are also in excellent agreement with experimental results, with all features accounted for.

**Table 3:** Experimental and simulated Raman spectral features (in cm^–1^) of β-TBB with comparison to published observations. Shoulder features are indicted with “sh”. Calculated intensities have been normalized to 1 in this spectral range

White, *et al.*^36^	Zakharov, *et al.*^12^	Burgos & Bonadeo^37^	Current work			
Experimental (ambient)	Experimental (ambient)	Experimental (100 K)	Experimental (291 K)	Experimental (105 K)	Calculated (100 K lattice)	Calculated intensities
—	—	17.5	15.5	17.1	18.7	0.969
—	—	21.5	21.8	22.1	23.9	0.923
—	—	38.5	—	39.0	40.7	0.105
—	42	44.5	40.6	44.4	45.0	0.637
—	—	46	—	46.2 (sh)	45.5	0.480
—	45.5	50.5	46.2	50.1	52.3	0.025
—	—	52	—	—	—	—
—	54	—	—	—	—	—
	—	62.0	—	—	—	—
—	87	—	—	—	—	—
125	126.4	—	126.4	126.6	125.6	0.312
—	—	—	—	128.5 (sh)	129.4	0.043
—	—	—	—	204 (sh)	200.0	0.024
203	203.6	—	203.6	206.7	201.6	0.062
—	—	—	209.0	209.3	205.2	0.039
208	209	—			205.5	0.127
220	221	—	221.4	223.4	224.4	1.000
—	—	—	—	224.5 (sh)	224.7	0.427
—	234	—	—	—	—	—

**Table 4:** Experimental and simulated Raman spectral features (in cm^–1^) of γ-TBB with comparison to published observations. Calculated intensities have been normalized to 1 in this spectral range

Burgos & Bonadeo^37^	Current work			
Experimental (330 K)	Experimental (320 K)	Experimental (118 K)	Calculated (332 K lattice)	Calculated intensities
14.5	14.5	14.8	14.6	1.000
28.0	27.8	28.2	29.5	0.502
			31.3	0.266
34.0	34.5	34.9	35.1	0.270
40.0	39.9	41.7	39.0	0.301
			42.0	0.054
—	126.5	126.8	124.7	0.171
—	—	—	129.1	0.001
—	205.8	205.4	198.8	0.018
—			200.4	0.001
—	208.9	209.7	204.4	0.023
—			204.5	0.014
—	222.1	222.3	223.6	0.280
—			223.9	0.093

#### iii. Polymorph stabilities

The results of energetic calculations revealed that β-TBB is 0.79 kJ mol^–1^ (per molecule) lower in total electronic energy than γ-TBB. Inclusion of zero-point energies from the vibrational simulations did not change this rank, with β-TBB remaining the more stable polymorph by 0.75 kJ mol^–1^ at 0 K. This is consistent with the observed dominance of β-TBB at temperatures below 307 K. Additional insight into the relative stabilities can be gained by decomposing the total energy into its conformational and cohesive components, which are listed in Table 5. β-TBB was found to have a more stable conformational energy, while γ-TBB had a more favorable cohesive energy.

**Table 5:** Calculated total electronic, conformational, and cohesive energies of the TBB polymorphs. All energies are in kJ mol^–1^ per molecule and corrected for BSSE

	Total energy (relative)	Conformational energy (relative)	Cohesive energy
β-TBB	0	0	–144.854
γ-TBB	0.791	2.111	–146.174

Temperature-dependent Gibbs free energy curves (also corrected for BSSE) were constructed for both polymorphs. β-TBB is more stable at 0 K by 0.75 kJ mol^–1^, but the curves cross at 76 K and γ-TBB becomes the more stable polymorph. While the transition temperature is quantitatively incorrect, it qualitatively agrees with experimental observations of γ-TBB being favored at higher temperatures.

#### iv. Normal mode eigenvector analysis

To examine the behavior of individual vibrations, the sub-100 cm^–1^ modes in the Raman spectra were further analyzed using ss-DFT to construct potential energy surfaces associated with their motions. The modes from the simulated spectra at 18.7, 40.7, and 45.0 cm^–1^ represent three different molecular rigid rotations within the β-TBB crystal about the crystallographic *a*, *b*, and *c* axes. The modes at 23.9, 52.3, and 45.5 cm^–1^ represent the same types of molecular motions, but with out-of-phase relationships between the members of the *Z* = 2 crystallographic unit cell, instead of the in-phase relationship found in the previous modes.

**Fig. 7:**
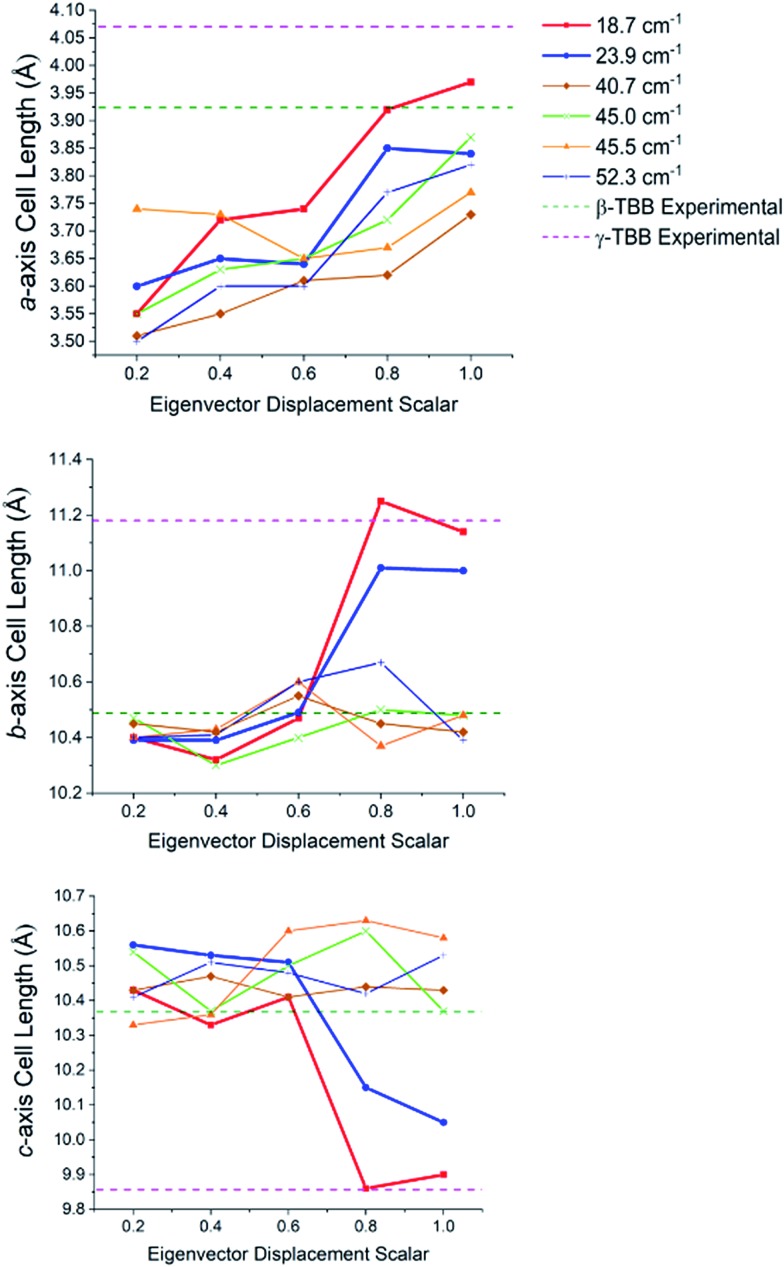
Changes in the lattice dimensions of β-TBB resulting from displacement along rotational-type lattice vibrations.

In order to test which (if any) of the modes were responsible for polymorph conversion, the atoms in the optimized β-TBB structure were incrementally displaced along the rotational mode eigenvectors and the unit cell was optimized around the new structure. The results of these eigenvector scans are shown in Fig. 7 and reveal that both the 18.7 cm^–1^ mode and the 23.9 cm^–1^ mode displaced structures optimized towards γ-TBB unit cell dimensions, with the 18.7 cm^–1^ mode results noticeably closer to the known γ-TBB values.

**Fig. 8:**
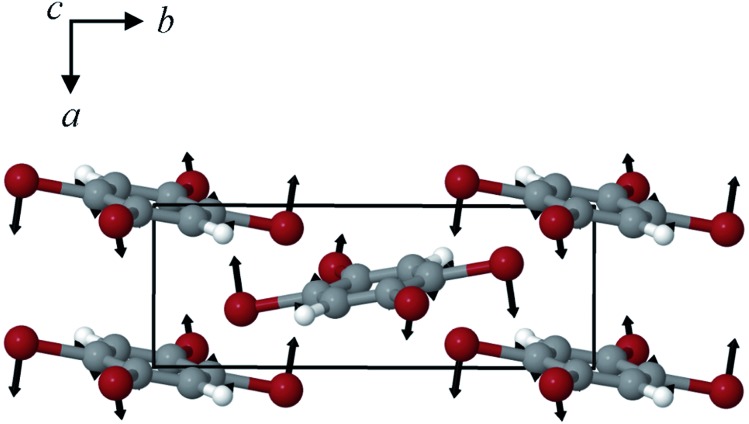
Calculated eigenvector representation of the 18.7 cm^–1^ vibrational mode in β-TBB.

By examining the eigenvector displaced crystals, it was found that the structure resulting from displacement along the 18.7 cm^–1^ eigenvector coordinate correlated very well with the atomic positions found in γ-TBB. This was not true for the out-of-phase 23.9 cm^–1^ mode, which forced TBB to an unstable high energy arrangement that did not correspond to either TBB polymorph. Although it appears from Fig. 7 that the 23.9 cm^–1^ mode goes towards γ-TBB formation, the actual atomic positioning contradicts this, and only the 18.7 cm^–1^ mode yields both unit cell dimensions and atomic positions that match those of γ-TBB. The vibrational motion of the 18.7 cm^–1^ mode is represented in Fig. 8.

A less strained and therefore more realistic approach for mapping the potential energy surface of the polymorph transformation coordinate is to freeze a single coordinate and then step along it, allowing the rest of the system to fully relax. A change in the intermolecular C2–Br2···C3′–C1 dihedral angle mimics the TBB ring tilting differences between the polymorphs that can be seen in Fig. 1 and this dihedral angle also corresponds to the rotational motion that forms the basis of the 18.7 cm^–1^ vibration.

Optimizations were performed by initially freezing the dihedral value at 10.5°, corresponding to the angle observed in a full optimization of β-TBB, and incrementally increasing the angle towards the γ-TBB polymorph. All other atomic positions and lattice parameters were allowed to energetically relax. A second energy minimum was found for the structure at a dihedral angle of 25.3°, which is identical to the dihedral angle observed in γ-TBB.

Although the unit cell dimensions and atomic positions are in good agreement with the γ-TBB structure at this dihedral angle, there was a 4.6% contraction of the cell volume as compared to experiment.^22^ However, contraction is expected in a 0 K structural simulation (especially *versus* the 332 K experimental data^22^) and the volume reduction is not unlike that reported for β-TBB when comparing room temperature to 100 K (ref. 21) structures, which is about 5.0%.

**Fig. 9:**
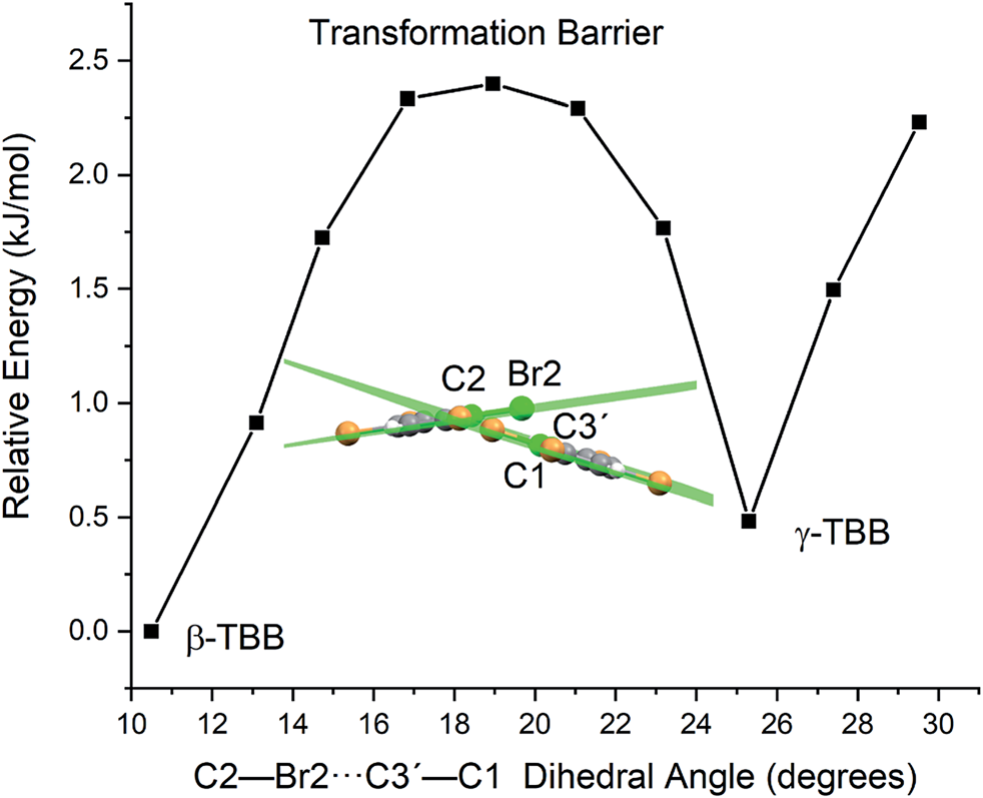
Potential energy surface for a frozen dihedral scan corresponding to the calculated 18.7 cm^–1^ mode.

The energy values determined over the course of the dihedral scanning are shown in Fig. 9. This revealed a barrier height of 2.40 kJ mol^–1^, which is a reasonable energy for the β → γ polymorph transformation considering the reported transition temperature of 307 K (ref. 12) (2.55 kJ mol^–1^). Performing a similar analysis on the dihedral angle changes that corresponded to the 23.9 cm^–1^ mode only resulted in an increase in energy along the potential energy scan, with no indication of γ-TBB formation. As a final check to ensure that the dihedral constraint placed on the γ-TBB did not cause chemically unrealistic approximations, a frequency analysis was performed and yielded no imaginary modes. These results collectively validate the dihedral-locked γ-TBB model solid to be a physically reasonable structure, and that the 18.7 cm^–1^ mode (observed experimentally at 17.1 cm^–1^ at 105 K and 15.5 cm^–1^ at 291 K) is the sole gateway lattice vibration responsible for polymorph conversion.

## IV. Conclusions

A combined experimental and computational investigation of the Raman spectra and solid-state vibrational modes of crystalline TBB has revealed the specific vibrational mode responsible for polymorph conversion between the β and γ polymorphs. The solid-state DFT simulations were in excellent agreement with the experimental measurements, allowing for a very accurate assessment of the vibrational modes. By considering the rotational-type molecular motions and scanning along their eigenvectors in β-TBB, it was found that the 17.1 cm^–1^ mode in the 105 K Raman spectrum (calculated at 18.7 cm^–1^ and experimentally observed at 15.5 cm^–1^ at 291 K) was able to act as a gateway vibration and induce the transformation of the β-form to the γ-form. This investigation also determined the barrier to conversion to be 2.40 kJ mol^–1^, which is consistent with the mild thermal energy required to form the γ-TBB polymorph. As suggested by previous reports,^6^,^12^ motion along this low-energy vibrational coordinate is translated into a macroscopic mechanical effect due to a combination of structural elasticity, anisotropic strain accompanying the transition, and the consequent stressed domain boundaries between β-form and γ-form domains within the crystal lattice. The discovery and characterization of the gateway vibration that is the foundation of polymorphic change in crystalline TBB offers new insights into its unusual thermosalient behavior and presents a compelling target of study in other materials that undergo temperature-induced polymorph changes.

## Conflicts of interest

There are no conflicts to declare.

## Supplementary Material

Supplementary information
