# Supplementary material for: Mapping the polymorphic transformation gateway vibration in crystalline 1,2,4,5-tetrabromobenzene
Source: Chem Sci. 2018 Nov 23;10(5):1332–41. doi: 10.1039/c8sc03897j (PMC6354909; doi:10.1039/c8sc03897j)
Supplement: Supplementary file 1 [file SC-010-C8SC03897J-s001.pdf]

Electronic Supplementary Material (ESI) for Chemical Science.
This journal is © The Royal Society of Chemistry 2018

Mapping the Polymorphic Transformation Gateway Vibration in Crystalline 1,2,4,5-Tetrabromobenzene

Adam J. Zaczek[a], Luca Catalano[b], Panče Naumov[b], and Timothy M. Korter*[a]

[a] Department of Chemistry, Syracuse University, 1-014 Center for Science and Technology, Syracuse, New York, 13244-4100, United States. E-mail: tmkorter@syr.edu

[b] New York University Abu Dhabi, P.O. Box 129188, Abu Dhabi, United Arab Emirates.

Table of Contents

Figure S1. Raman spectra for a pellet containing a majority of β-TBB with γ-TBB contamination over a range of temperatures. Pure powder Raman data for both TBB polymorphs (scaled for clarity) are included to explicitly show the source of contamination from γ-TBB and its spectral shifting with temperature change.

Figure S2. Raman spectra for a pellet containing a majority of γ-TBB with β-TBB contamination over a range of temperatures. Pure powder Raman data for both TBB polymorphs (scaled for clarity) are included to explicitly show the source of contamination from β-TBB and its spectral shifting with temperature change.

Table S1. Solid-state DFT (PBE-D3/def2-TZVP) simulated vibrational frequencies for β-TBB. Frequency is listed in cm^{-1}, IR intensities are listed in units of km/mol, and Raman intensities are listed in arbitrary units, normalized to 1.

Table S2. Solid-state DFT (PBE-D3/def2-TZVP) simulated vibrational frequencies for γ-TBB. Frequency is listed in cm^{-1}, IR intensities are listed in units of km/mol, and Raman intensities are listed in arbitrary units, normalized to 1.

Table S3. Comparison of the intermolecular atomic distances from the optimized TBB structures and the 18.7, 23.9, 40.7, 45.0, 45.5, and 52.3 cm^{-1} eigenvectors with a displacement scalar of 1.0.

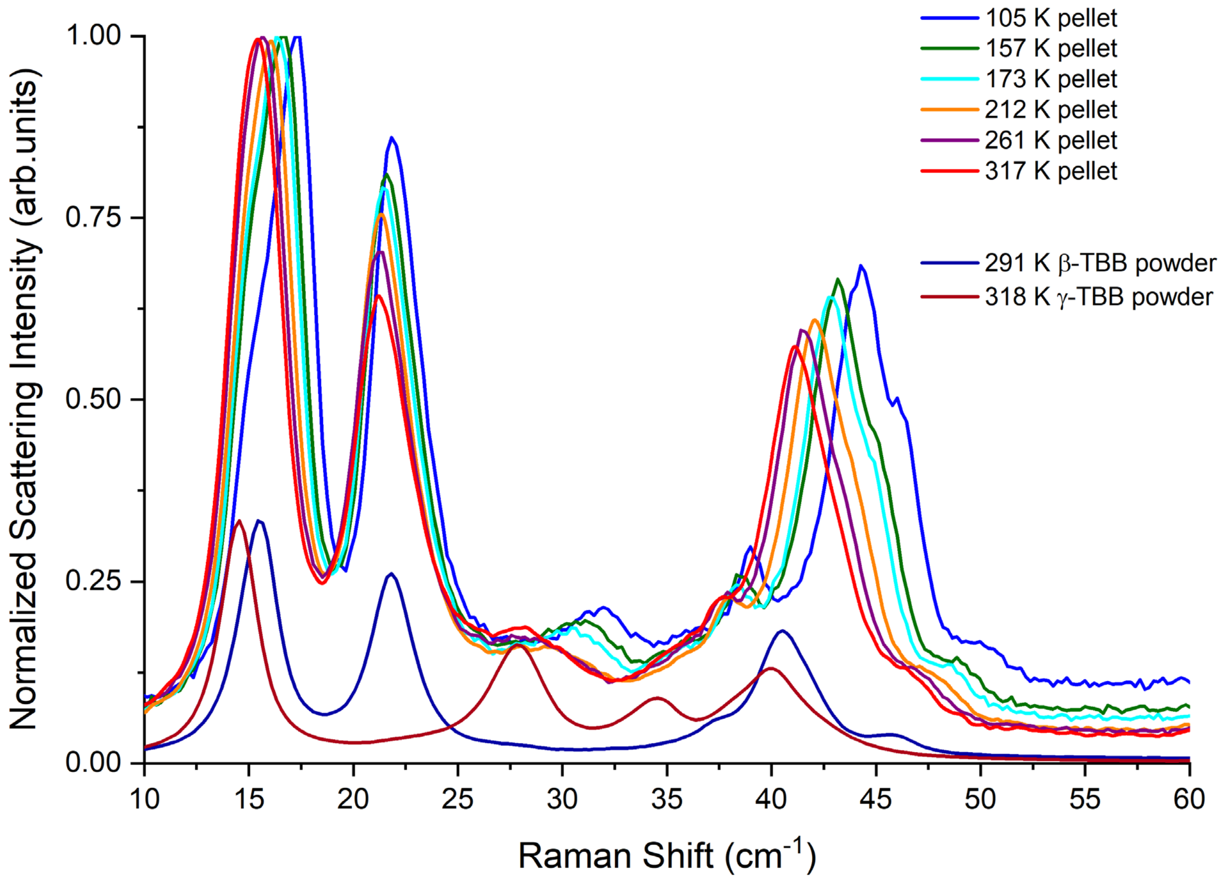

Figure S1. Raman spectra for a pellet containing a majority of β-TBB with γ-TBB contamination over a range of temperatures. Pure powder Raman data for both TBB polymorphs (scaled for clarity) are included to explicitly show the source of contamination from γ-TBB and its shifting with temperature change.

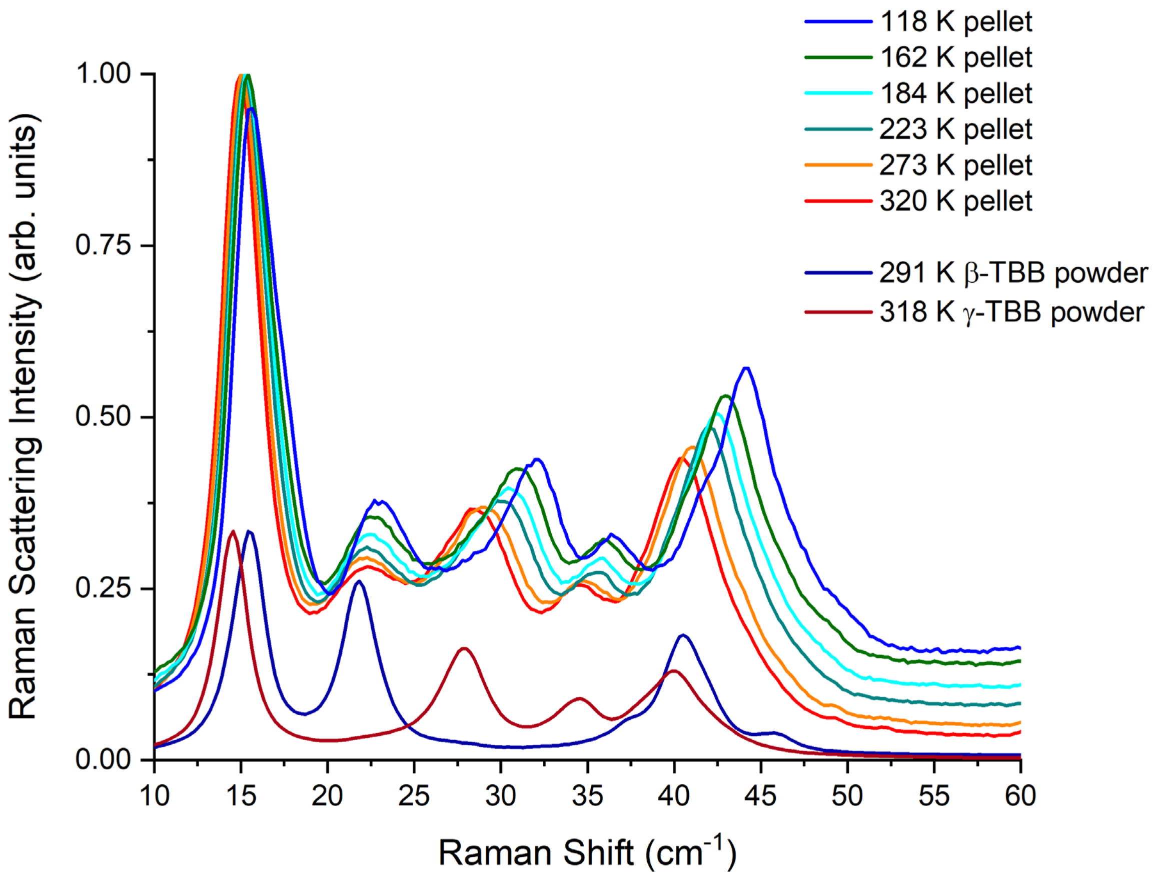

Figure S2. Raman spectra for a pellet containing a majority of γ-TBB with β-TBB contamination over a range of temperatures. Pure powder Raman data for both TBB polymorphs (scaled for clarity) are included to explicitly show the source of contamination from β-TBB and its spectral shifting with temperature change.

Table S1. Solid-state DFT (PBE-D3/def2-TZVP) simulated vibrational frequencies for β-TBB. Frequency is listed in cm^{-1}, IR intensities are listed in units of km/mol, and Raman intensities are listed in arbitrary units, normalized to 1.

Frequency (cm^{-1})	IR Intensity (km/mol)	Raman Intensity (arb. units)	Frequency (cm^{-1})	IR Intensity (km/mol)	Raman Intensity (arb. units)
18.667	0.000	0.969	572.089	0.290	0.000
23.892	0.000	0.923	572.107	0.030	0.000
31.901	0.010	0.000	658.869	0.000	0.546
40.655	0.000	0.105	662.600	0.000	0.014
45.033	0.000	0.637	671.309	0.000	0.009
45.525	0.000	0.480	672.176	0.000	0.011
51.646	0.100	0.000	779.369	0.000	0.009
52.301	0.000	0.025	791.489	0.000	0.019
54.550	0.030	0.000	856.412	0.000	0.001
59.365	0.190	0.000	857.100	0.000	0.006
61.103	0.640	0.000	870.813	19.510	0.000
110.564	0.530	0.000	871.116	113.750	0.000
113.294	0.160	0.000	1001.336	123.670	0.000
125.584	0.000	0.312	1002.458	180.020	0.000
129.355	0.000	0.043	1099.538	71.930	0.000
133.507	0.370	0.000	1100.451	52.050	0.000
135.913	0.450	0.000	1116.254	0.000	0.033
146.477	1.770	0.000	1117.369	0.000	0.018
148.787	0.910	0.000	1229.086	0.000	0.014
199.969	0.000	0.024	1234.571	0.000	0.003
201.553	0.000	0.062	1294.313	87.560	0.000
205.188	0.000	0.039	1294.324	95.620	0.000
205.487	0.000	0.127	1307.573	5.210	0.000
224.402	0.000	1.000	1308.157	0.620	0.000
224.722	0.000	0.427	1411.610	258.060	0.000
316.004	0.000	0.011	1412.832	165.600	0.000
316.198	0.000	0.001	1514.721	0.000	0.003
384.177	9.710	0.000	1515.194	0.000	0.030
384.843	12.730	0.000	1528.296	0.000	0.268
430.178	7.140	0.000	1540.082	0.000	0.007
430.669	38.560	0.000	3123.173	150.640	0.000
451.543	0.000	0.011	3123.417	185.050	0.000
452.327	0.000	0.013	3124.796	0.000	0.280
519.324	13.360	0.000	3125.996	0.000	0.152
520.228	24.350	0.000			

Table S2. Solid-state DFT (PBE-D3/def2-TZVP) simulated vibrational frequencies for γ-TBB. Frequency is listed in cm^{-1}, IR intensities are listed in units of km/mol, and Raman intensities are listed in arbitrary units, normalized to 1.

Frequency (cm^{-1})	IR Intensity (km/mol)	Raman Intensity (arb. units)	Frequency (cm^{-1})	IR Intensity (km/mol)	Raman Intensity (arb. units)
14.569	0.000	1.000	574.931	0.170	0.000
29.397	0.250	0.000	575.165	0.010	0.000
29.516	0.000	0.502	657.815	0.000	0.100
31.347	0.000	0.266	660.975	0.000	0.002
35.093	0.000	0.270	676.255	0.000	0.001
39.033	0.000	0.301	676.916	0.000	0.001
41.980	0.000	0.054	778.380	0.000	0.001
43.954	0.100	0.000	789.955	0.000	0.000
44.916	0.160	0.000	860.020	0.000	0.002
48.253	0.000	0.000	860.850	0.000	0.000
52.520	0.170	0.000	872.999	99.470	0.000
114.016	0.020	0.000	873.627	10.310	0.000
116.046	0.000	0.000	999.710	141.520	0.000
124.720	0.000	0.171	1001.257	154.200	0.000
129.053	0.000	0.001	1095.094	75.940	0.000
134.778	0.860	0.000	1096.066	66.630	0.000
135.692	0.890	0.000	1112.789	0.000	0.014
142.613	0.010	0.000	1113.822	0.000	0.003
142.754	0.160	0.000	1226.249	0.000	0.001
198.788	0.000	0.018	1231.211	0.000	0.000
200.400	0.000	0.023	1293.142	101.360	0.000
204.418	0.000	0.000	1293.720	93.220	0.000
204.523	0.000	0.014	1302.355	6.440	0.000
223.614	0.000	0.280	1302.704	2.150	0.000
223.936	0.000	0.093	1409.325	218.050	0.000
318.343	0.000	0.003	1409.408	223.670	0.000
318.871	0.000	0.001	1512.079	0.000	0.002
382.173	10.000	0.000	1512.651	0.000	0.008
383.217	13.270	0.000	1525.895	0.000	0.049
433.625	35.840	0.000	1536.985	0.000	0.000
433.795	3.330	0.000	3122.372	131.040	0.000
449.729	0.000	0.000	3122.422	121.280	0.000
451.105	0.000	0.002	3123.719	0.000	0.045
518.497	24.860	0.000	3124.593	0.000	0.024
518.911	23.000	0.000			

Table S3. Comparison of the intermolecular atomic distances from the optimized TBB structures and the 18.7, 23.9, 40.7, 45.0, 45.5, and 52.3 cm^{-1} eigenvectors with a displacement scalar of 1.0.

	C1—C2	C1—C3′	C2—C3	C1—Br1	C2—Br2
β-TBB Optimized	1.402	1.396	1.395	1.895	1.896
γ-TBB Optimized	1.371	1.406	1.399	1.8733	1.904
18.7 cm^{-1} displaced mode	1.401	1.398	1.397	1.881	1.904
23.9 cm^{-1} displaced mode	1.402	1.401	1.393	1.894	1.904
40.7 cm^{-1} displaced mode	1.402	1.401	1.393	1.892	1.903
45.0 cm^{-1} displaced mode	1.402	1.401	1.393	1.891	1.903
45.5 cm^{-1} displaced mode	1.403	1.402	1.392	1.891	1.904
52.3 cm^{-1} displaced mode	1.402	1.401	1.393	1.891	1.904
